# Association between glucokinase regulator gene polymorphisms and serum uric acid levels in Taiwanese adolescents

**DOI:** 10.1038/s41598-022-09393-5

**Published:** 2022-04-01

**Authors:** Li-Ju Ho, Chieh-Hua Lu, Ruei-Yu Su, Fu-Huang Lin, Sheng-Chiang Su, Feng-Chih Kuo, Nain-Feng Chu, Yi-Jen Hung, Jhih-Syuan Liu, Chang-Hsun Hsieh

**Affiliations:** 1 Graduate Institute of Clinical Medicine, College of Medicine, Taipei Medical University, Taipei, Taiwan, ROC; 2 Division of Endocrinology and Metabolism, Department of Internal Medicine, Tri-Service General Hospital, National Defense Medical Center, No. 325, Section 2, Cheng-Kung Road, Neihu District, Taipei City, 11490 Taiwan, ROC; 3 Division of Clinical Pathology, Department of Pathology, Tri-Service General Hospital, National Defense Medical Center, Taipei, Taiwan, ROC; 4 Department of Pathology and Laboratory Medicine, Taoyuan Armed Forces General Hospital, Taoyuan, Taiwan, ROC; 5 School of Public Health, National Defense Medical Center, Taipei, Taiwan, ROC

**Keywords:** Genetics, Endocrinology, Rheumatology, Risk factors

## Abstract

The glucokinase regulator gene (*GCKR*) is located on chromosome 2p23. It plays a crucial role in maintaining plasma glucose homeostasis and metabolic traits. Recently, genome-wide association studies have revealed a positive association between hyperuricemia and *GCKR* variants in adults. This study investigated this genetic association in Taiwanese adolescents. Data were collected from our previous cross-sectional study (Taipei Children Heart Study). The frequencies of various genotypes (CC, CT, and TT) or alleles (C and T) of the *GCKR* intronic single-nucleotide polymorphism (SNP) rs780094 and the coding SNP rs1260326 (Pro446Leu, a common 1403C-T transition) were compared between a total of 968 Taiwanese adolescents (473 boys, 495 girls) with hyperuricemia or normal uric acid levels on the basis of gender differences. Logistic and linear regression analyses explored the role of *GCKR* in abnormal uric acid (UA) levels. Boys had higher UA levels than girls (6.68 ± 1.29 and 5.23 ± 0.95 mg/dl, respectively, *p* < 0.001). The analysis of both SNPs in girls revealed that the T allele was more likely to appear in patients with hyperuricemia than the C allele. After adjusting for confounders, the odds ratio (OR) for hyperuricemia incidence in the TT genotype was 1.75 (95% confidence interval [CI] 1.02–3.00), which was higher than that in the C allele carriers in rs1260326 in the girl population. Similarly, the TT genotypes had a higher risk of hyperuricemia, with an OR of 2.29 (95% CI 1.11–4.73) for rs1260326 and 2.28 (95% CI 1.09–4.75) for rs780094, than the CC genotype in girl adolescents. The T (Leu446) allele of *GCKR* rs1260326 polymorphism is associated with higher UA levels in Taiwanese adolescent girls.

## Introduction

Uric acid (UA) is a by-product of purine metabolism in humans. Hyperuricemia is associated with an increased risk of gout and various chronic diseases such as hypertension, type 2 diabetes mellitus (T2DM), and cardiovascular disease (CVD)^1,2^. Moreover, hyperuricemia was shown to be an independent risk factor for all-cause and CVD mortalities^3^. Thus, it is pertinent to evaluate patients with hyperuricemia as early as possible.

The serum UA levels can be influenced by several lifestyle choices such as the intake of protein and alcohol^4^. The variation in the prevalence of hyperuricemia may be attributed to various lifestyle patterns. Furthermore, genes play a vital role in regulating the serum UA levels^5^. Serum UA levels are heritable and related to mutations in genes encoding the enzymes involved in the purine salvage pathway^6^. In recent decades, several case–control studies and genome-wide association studies (GWAS) have explored the function of genetic loci in hyperuricemia^5,7–9^. Several loci were identified to be associated with the serum UA levels, including the solute carrier family 2 member 9 (SLC2A9) and ATP-binding cassette subfamily G member 2 (ABCG2), both of which have been studied in different ethnic populations^10–17^. This association has also been noted in the adolescent population^18,19^. However, most of these candidate genes regulate the renal excretion of UA instead of regulating the endogenous UA production.

Glucokinase (GCK) is a physiological glucose sensor. Through glucose phosphorylation, it plays a crucial function in glucose-stimulated insulin release^20^. The GCK regulator gene (*GCKR*) is located on chromosome 2p23, contains 19 exons, and encodes a 68-kDa protein^21,22^. Mutations in this gene are associated with insulin signaling process-related disorders. Several studies have revealed an association between *GCKR* variants and metabolic syndrome and/or its components, such as the levels of fasting glucose and triglyceride (TG)^23–25^. However, the role of *GCKR* in regulating the serum UA levels remains unclear. Recently, several GWAS have shown that the common variants of *GCKR* are associated with hyperuricemia and replicate among various ethnic populations, including the T allele of the SNP rs780094 found in the New Zealand European, Polynesian, and Chinese populations^10,26,27^ and the risk T allele of the SNP rs1260326 found in the Chinese and Japanese populations^27–29^. Apart from the T allele, the A allele of rs780094 in GCKR has been consistently shown to be associated with the risk of gout in Chinese and Japanese populations^30,31^. However, a large meta-analysis of case–control studies showed no similar associations between the *GCKR* variants and serum UA levels^32^. Therefore, we sought to examine the apparent pleiotropic effect of *GCKR* polymorphisms on the serum UA levels.

It is imperative to note that most GWAS recruited adult populations. Compared with adolescents, adults are prone to having hyperuricemia-associated diets and lifestyle habits, which include diets/lifestyles related to obesity, high purine intakes, certain medication use, and alcoholic beverage consumption^33^. Moreover, the serum UA levels in children and adolescents change during development. The levels gradually increase from birth to the end of elementary school age and then increase considerably in boys and slightly in girls, resulting in a considerable difference in the serum UA levels between the genders^34^. The study explored the genetic association of the common functional variants of *GCKR* with the serum UA levels in Taiwanese adolescents because of the uniqueness of hyperuricemia in teenagers.

## Material and methods

### Study sample and ethics statement

The study population was previously enrolled in an epidemiological study to evaluate the obesity and CVD risk factors among school children in Taipei in 2006. The details of this previous study have been published previously^35,36^. A total of 968 children (473 boys and 495 girls) had available biochemical data, and their DNA samples were included in the final analyses. Informed consent was obtained from the participants and their parents before enrollment. This study was approved by the Institutional Review Board of the Tri-Service General Hospital (TSGHIRB No. 095-05-139 and TSGHIRB No. 095-05-013) and was conducted in accordance with the ethical principles of the Declaration of Helsinki, the Belmont Report, and International Ethical Guidelines for Health-related Research Involving Humans.

### Data collection and laboratory measurements

In the previous study, all participants completed a questionnaire detailing their age, gender, demographic characteristics, medical history, pubertal development, and lifestyle characteristics, including cigarette smoking and alcohol consumption. Body weight (BW) was measured using a ruler attached to a scale by a trained nurse. Body mass index (BMI) was calculated by dividing the BW (kg) by the square of the height (m). According to the definition provided by the World Health Organization and International Diabetes Federation, waist circumference was measured in the horizontal plane midway between the lowest ribs and iliac crest. After a 10-h fast, blood samples were collected and used to determine the levels of plasma glucose and lipids, including TG and high-density lipoprotein cholesterol (HDL-C). The serum UA levels were measured using the uricase/peroxidase method (DVIA1650-Autoanalyzer, Siemens Healthcare Diagnostics). After 10 min of rest in a seated position, blood pressure was measured on the right arm.

### Definition of hyperuricemia and grouping

The definition of hyperuricemia is gender-specific, with a serum UA level of ≥ 7 mg/dl for boys and ≥ 6 mg/dl for girls^37^. Therefore, the study participants were divided into two groups: hyperuricemia group (HUA) and normal UA group (NUA) with gender specification.

### DNA extraction and genotyping

DNA was isolated from blood samples using a QIAamp^®^ DNA Blood kit following the manufacturer’s instructions (Qiagen Inc., Hilden, Germany) during the previous study. The quality of the isolated genomic DNAs was assessed through agarose gel electrophoresis, and the DNAs were quantified using spectrophotometry. The SNPs were genotyped using TaqMan^®^ Genotyping assays. TaqMan^®^ probes and Universal PCR Master Mix were purchased from Applied Biosystems Inc. (ABI; Foster City, CA, USA). TaqMan^®^ PCR was performed according to the manufacturer’s standard protocol. Each sample underwent 40 amplification cycles through the ABI GeneAmp^®^ PCR System 9700 instrument. Fluorescent signals of the two probes, corresponding to the detection of the two alleles, were analyzed using the ABI PRISM^®^ 7900HT Sequence Detection System. Genotypes were automatically determined using the ABI Sequence Detection Software. Two SNPs within *GCKR* (rs1260326 and rs780094) were selected for analysis to validate the association between the genetic variants and hyperuricemia/gout and/or metabolic traits in previous studies^10,11,26–30,32,38–43^. Genotype distributions matched expectations under the Hardy–Weinberg equilibrium (*p* = 0.38 and 0.10 for rs1260326 and rs780094, respectively) with a calling rate of 100% in the participants.

### Statistical analyses

All analyses were performed using the Statistical Package for the Social Sciences software (IBM SPSS Statistics for Windows, Version 20.0; IBM Corp. Armonk, NY, USA: https://www.ibm.com/ support/pages/spss-statistics-20-available-download). The age, BW, body height, BMI, demographic characteristics, and laboratory values were described as means and standard deviations and compared between the two genders. Categorical data were presented as numbers (n) and percentages (%). The Kolmogorov–Smirnov test was used for distribution testing. An independent sample *t*-test was used to compare the means of the two independent groups; a chi-square test was used to compare the categorical variables.

The cohort was divided into subgroups on the basis of the alleles (T and C) and genotypes (TT, CT, and CC) of the two *GCKR* SNPs (rs780094 and rs1260326). The frequencies of alleles and genotypes were compared between HUA and NUA. The relationships between dominant and non-dominant allele carriers were also evaluated using contingency tables. The Hardy–Weinberg equilibrium was used to test the comparison using the chi-square goodness-of-fit test. A linear regression model was used to evaluate the associations of these SNPs with the serum UA levels. A logistic regression test was used to adjust for the possible confounders, including age, BMI, and metabolic syndrome components. Moreover, this test was used to evaluate the independent function of genetic polymorphism in regulating the abnormal serum UA levels. A logistic regression test was performed to calculate the odds ratios (ORs) with 95% confidence intervals (CIs) for the risk genotypes. Metabolic traits were compared between different *GCKR* variants. After adjusting age and sex, logistic regression analyses were performed to evaluate the independent role of *GCKR* genotypes in determining the metabolic traits. All of the abovementioned analyses were performed with conjoint analysis and gender specification. Statistical significance was defined as a *p*-value of < 0.05.

## Results

Table 1 presents the participant demographics. The mean age was 13.3 ± 1.0 years, and the mean BMI was 21.0 ± 3.9 kg/m^2^. Generally, girls had more favorable metabolic characteristics than boys. The serum UA levels were higher in boys than in girls (6.68 ± 1.29 and 5.23 ± 0.95 mg/dl, respectively, *p* < 0.001). In addition, the frequency of hyperuricemia was higher in boys (36.6%) than in girls (17.0%; *p* < 0.001). We noted that girls with hyperuricemia had a significantly higher T allele frequency than the C allele frequency in both SNPs (*p* = 0.028 and 0.034 in rs780094 and rs1260326, respectively). However, no similar difference was observed in boys (Supplementary Table 1). Furthermore, there was no difference in the genotype frequencies (CC, CT, and TT) of either SNP between HUA and NUA in either gender (Supplementary Table 2).

**Table 1:** Demographic characteristics of the study population.

Group (n)	All (968)	Boys (473)	Girls (495)	p
Age (years)	13.3 ± 1.0	13.3 ± 1.0	13.4 ± 1.0	0.136
FPG (mg/dl)	92.7 ± 6.7	93.8 ± 6.5	91.8 ± 6.8	< 0.001
BMI (kg/m^2^)	21.0 ± 3.9	21.6 ± 4.2	20.4 ± 3.4	< 0.001
WC (cm)	76.5 ± 9.8	78.8 ± 10.8	74.3 ± 8.2	< 0.001
SBP (mmHg)	114.8 ± 13.7	118.3 ± 14.3	111.6 ± 12.4	< 0.001
DBP (mmHg)	69.7 ± 10.0	69.3 ± 10.7	70.0 ± 9.4	0.293
HDL-C (mg/dl)	49.7 ± 11.4	47.5 ± 11.3	51.8 ± 11.2	< 0.001
TG (mg/dl)	71.5 ± 35.6	71.3 ± 40.0	71.7 ± 30.8	0.865
UA (mg/dl)	5.94 ± 1.34	6.68 ± 1.29	5.23 ± 0.95	< 0.001
HUA (n/%)	257 (26.5%)	173 (36.6%)	84 (17.0%)	< 0.001

FPG: fasting plasma glucose; BMI: body mass index; WC: waist circumference; SBP: systolic blood pressure; DBP: diastolic blood pressure; HDL-C: high-density lipoprotein cholesterol; TG: triglyceride; UA: uric acid; HUA: hyperuricemia.

Table 2 shows the values of metabolic traits among the various genotypes of the *GCKR* variants (rs1260326 and rs780094). In *GCKR* rs1260326, the participants with the TT genotype had lower HDL-C and higher serum TG levels than those with the TC and CC genotypes (*p* = 0.039 and *p* < 0.001, respectively). A significant difference was maintained with gender specification, except for HDL-C levels in the girl population. On the other hand, in *GCKR* rs780094, the participants with the TT genotype had lower HDL-C and higher serum TG levels than those with the TC and CC genotypes (*p* = 0.037 and *p* = 0.001, respectively). However, this effect was observed only in the boy population. The CT genotype was associated with the highest UA levels, followed by the TT and CC genotypes, in both SNPs in the entire population (*p* = 0.028 in rs1260326 and *p* = 0.035 in rs780094, respectively). However, the serum UA levels showed no significant difference in both genders.

**Table 2:** Metabolic characteristics according to GCKR rs1260326 and rs780094 genotypes.

GCKR rs1260326	CC	CT	TT	p value
**All**				
WC	75.6 ± 9.6	76.7 ± 9.5	76.8 ± 10.4	0.326
TG	64.8 ± 27.4	70.9 ± 33.6	77.9 ± 43.6	< 0.001*
HDL-C	51.4 ± 11.4	49.4 ± 11.6	48.8 ± 11.1	0.039*
SBP	112.9 ± 13.3	115.8 ± 13.5	114.6 ± 14.3	0.037*
DBP	69.0 ± 9.5	69.9 ± 10.2	69.7 ± 10.1	0.538
FPG	92.8 ± 6.3	92.9 ± 6.8	92.4 ± 6.8	0.628
UA	5.73 ± 1.28	6.02 ± 1.35	5.96 ± 1.36	0.028*
**Boys**				
WC	77.4 ± 10.5	78.9 ± 10.3	79.3 ± 11.9	0.419
TG	63.1 ± 27.7	69.4 ± 36.5	80.9 ± 51.5	0.003*
HDL-C	50.1 ± 12.7	47.0 ± 10.8	46.5 ± 11.1	0.047*
SBP	116.8 ± 13.8	119.0 ± 13.9	117.8 ± 15.2	0.429
DBP	68.7 ± 10.6	69.6 ± 10.5	69.1 ± 11.2	0.753
FPG	93.1 ± 5.5	93.8 ± 6.8	94.2 ± 6.5	0.465
UA	6.56 ± 1.24	6.73 ± 1.29	6.66 ± 1.32	0.563
**Girls**				
WC	74.3 ± 8.7	74.2 ± 8.0	74.4 ± 8.2	0.958
TG	66.0 ± 27.2	72.6 ± 30.2	75.1 ± 34.4	0.047*
HDL-C	52.2 ± 10.4	52.0 ± 11.9	50.9 ± 10.7	0.601
SBP	110.2 ± 12.2	112.2 ± 12.2	111.6 ± 12.8	0.322
DBP	69.2 ± 8.7	70.3 ± 10.0	70.3 ± 9.0	0.569
FPG	92.6 ± 6.8	91.9 ± 6.7	90.7 ± 6.6	0.066
UA	5.15 ± 0.93	5.23 ± 0.92	5.29 ± 1.01	0.464

GCKRrs780094	CC	CT	TT	p value
**All**				
WC	75.6 ± 9.4	76.7 ± 9.8	76.7 ± 10.2	0.305
TG	64.7 ± 27.1	71.8 ± 35.5	77.5 ± 41.7	0.001*
HDL-C	51.4 ± 11.4	49.2 ± 11.5	49.1 ± 11.2	0.037*
SBP	112.9 ± 13.2	115.8 ± 13.7	114.5 ± 14.1	0.029*
DBP	68.9 ± 9.6	70.0 ± 10.3	69.7 ± 10.0	0.435
FPG	92.9 ± 6.5	92.9 ± 6.7	92.3 ± 6.9	0.466
UA	5.74 ± 1.27	6.01 ± 1.35	5.97 ± 1.36	0.035*
**Boys**				
WC	77.4 ± 10.4	79.1 ± 10.6	79.1 ± 11.5	0.409
TG	62.6 ± 27.0	70.6 ± 39.2	80.5 ± 49.1	0.006*
HDL-C	50.3 ± 12.5	46.7 ± 10.8	46.8 ± 11.1	0.025*
SBP	116.7 ± 13.5	119.0 ± 14.1	117.8 ± 15.2	0.374
DBP	68.9 ± 10.7	69.8 ± 10.6	68.6 ± 10.9	0.564
FPG	93.5 ± 5.9	93.7 ± 6.7	94.2 ± 6.5	0.654
UA	6.58 ± 1.23	6.70 ± 1.30	6.72 ± 1.32	0.680
**Girls**				
WC	74.3 ± 8.5	74.1 ± 8.0	74.5 ± 8.3	0.899
TG	66.2 ± 27.2	73.1 ± 30.9	74.9 ± 33.8	0.051
HDL-C	52.2 ± 10.5	51.9 ± 11.8	51.1 ± 10.9	0.726
SBP	110.3 ± 12.4	112.3 ± 12.3	111.7 ± 12.5	0.329
DBP	69.0 ± 8.7	70.2 ± 9.9	70.7 ± 9.0	0.315
FPG	92.5 ± 6.9	92.0 ± 6.7	90.5 ± 6.8	0.045*
UA	5.15 ± 0.93	5.24 ± 0.93	5.30 ± 1.01	0.407

WC: waist circumstance (cm); TG: triglyceride (mg/dl); HDL-C: high-density lipoprotein cholesterol (mg/dl); SBP: systolic blood pressure (mmHg); DBP: diastolic blood pressure (mmHg); FPG: fasting plasma glucose (mg/dl); UA: uric acid (mg/dl).

*Statistically significant differences.

Table 3 summarizes the results of the linear regression analyses of the association of two *GCKR* polymorphisms (rs1260326 and rs780094) with the serum UA levels. The beta values for the serum UA level were calculated using linear regression and adjusted for several confounders. In the overall group, the T carriers with the *GCKR* polymorphism rs1260326 were associated with a mean UA level change of 0.27 mg/dl compared with those with the CC genotype (95% CI 0.07–0.48, *p* = 0.009); the T carriers with the *GCKR* polymorphism rs780094 were associated with a mean UA level change of 0.26 mg/dl compared with those with the CC genotype (95% CI 0.06–0.46, *p* = 0.010). However, after adjusting for confounders, the T carriers showed no significant increase in the serum UA levels compared with those with the CC genotype in the overall group and in both genders for either *GCKR* polymorphism, rs1260326 or rs780094.

**Table 3:** Linear regression analyses of uric acid level in Taiwanese children by GCKR gene genotypes (rs1260326, rs780094).

	Model 1		Model 2	
	Beta (95% CI)	P value	Beta (95% CI)	P value
**All**				
GCKRrs1260326				
Additive model	0.11 (− 0.13 to 0.23)	0.08	0.02 (− 0.07 to 0.12)	0.627
TT vs (CC + CT)	0.03 (− 0.16 to 0.22)	0.759	− 0.01 (− 0.16 to 0.14)	0.880
(CT + TT) vs CC	0.27 (0.07 to 0.48)*	0.009	0.08 (− 0.08 to 0.24)	0.328
GCKRrs780094				
Additive model	0.12 (− 0.01 to 0.24)	0.063	0.04 (− 0.06 to 0.13)	0.412
TT vs (CC + CT)	0.05 (− 0.15 to 0.25)	0.644	0.03 (− 0.12 to 0.18)	0.706
(CT + TT) vs CC	0.26 (0.06 to 0.46)*	0.010	0.08 (− 0.08 to 0.23)	0.340
**Boys**				
GCKRrs1260326				
Additive model	0.04 (− 0.14 to 0.21)	0.666	− 0.05 (− 0.20 to 0.11)	0.549
TT vs (CC + CT)	− 0.03 (− 0.29 to 0.24)	0.849	− 0.12 (− 0.36 to 0.12)	0.316
(CT + TT) vs CC	0.15 (− 0.15 to 0.45)	0.339	0.02 (− 0.25 to 0.28)	0.911
GCKRrs780094				
Additive model	0.07 (− 0.11 to 0.25)	0.440	− 0.02 (− 0.18 to 0.14)	0.788
TT vs (CC + CT)	0.05 (− 0.22 to 0.33)	0.700	− 0.05 (− 0.30 to 0.19)	0.674
(CT + TT) vs CC	0.13 (− 0.16 to 0.42)	0.387	0.001 (− 0.26 to 0.26)	0.996
**Girls**				
GCKRrs1260326				
Additive model	0.07 (− 0.04 to 0.19)	0.219	0.06 (− 0.05 to 0.17)	0.271
TT vs (CC + CT)	0.09 (− 0.10 to 0.28)	0.354	0.07 (− 0.11 to 0.25)	0.452
(CT + TT) vs CC	0.11 (− 0.09 to 0.30)	0.272	0.10 (− 0.08 to 0.28)	0.291
GCKRrs780094				
Additive model	0.08 (− 0.04 to 0.20)	0.183	0.06 (− 0.05 to 0.18)	0.257
TT vs (CC + CT)	0.10 (− 0.10 to 0.29)	0.318	0.07 (− 0.12 to 0.25)	0.474
(CT + TT) vs CC	0.11 (− 0.08 to 0.30)	0.236	0.10 (− 0.08 to 0.28)	0.257

Model 1: unadjusted analysis for possible confounding factors, Model 2: adjusted for age, body mass index, waist circumference, blood pressure, fasting plasma glucose, triglycerides, high density lipoprotein cholesterol, and gender (only in overall group).

CI: confidence interval.

*Statistically significant differences.

Table 4 shows the association between the *GCKR* variants and hyperuricemia, which was identified using logistic regression analyses. The conjoint analysis showed that the CT and TT genotypes were associated with a higher risk of hyperuricemia than the CC genotypes. However, only the TT genotype showed statistical significance in both SNPs after adjusting for confounders, with an OR of 1.82 (95% CI 1.13–2.92, *p* = 0.014) for rs1260326 and 1.87 (95% CI 1.16–3.01, *p* = 0.01) for rs780094. The effects were also noted in additive and dominant models. Only the results obtained from the girl population remained statistically significant after adjusting for confounders, with an OR of 2.29 (95% CI 1.11–4.73, *p* = 0.026) for rs1260326 and 2.28 (95% CI 1.09–4.75, *p* = 0.028) for rs780094. Furthermore, participants with the TT genotype had a higher risk of hyperuricemia, with an OR of 1.75 (95% CI 1.02–3.00, *p* = 0.041), than those with the C allele of rs1260326 in the girl group.

**Table 4:** Logistic regression analyses of hyperuricemia in Taiwanese children by GCKR gene genotypes (rs1260326、rs780094).

	Genotype	NUA (n, %)	HUA (n, %)	Genetic model	Model 1			Model 2		
					OR	95%CI	p value	OR	95%CI	p value
**All**										
GCKRrs1260326	CC	172 (24.3)	41 (16.0)		Reference			Reference		
	CT	360 (50.8)	135 (25.5)		1.57*	1.06 to 2.33	0.024	1.41	0.92 to 2.18	0.116
	TT	176 (24.9)	81 (31.5)		1.93*	1.26 to 2.97	0.008	1.82*	1.13 to 2.92	0.014
				Additive	1.37*	1.11 to 1.68	0.003	1.34*	1.06 to 1.69	0.014
				Recessive	1.39*	1.02 to 1.90	0.039	1.41	0.99 to 2.00	0.056
				Dominant	1.69*	1.16 to 2.46	0.006	1.54*	1.02 to 2.33	0.04
GCKRrs780094	CC	182 (25.6)	43 (16.7)		Reference			Reference		
	CT	369 (52.0)	140 (54.5)		1.61*	1.09 to 2.36	0.016	1.45	0.95 to 2.21	0.085
	TT	159 (22.4)	74 (28.8)		1.97*	1.28 to 3.03	0.002	1.87*	1.16 to 3.01	0.01
				Additive	1.39*	1.12 to 1.71	0.002	1.36*	1.07 to 1.72	0.011
				Recessive	1.4*	1.02 to 1.94	0.04	1.43	1.00 to 2.05	0.053
				Dominant	1.72*	1.19 to 2.48	0.004	1.57*	1.05 to 2.35	0.028
**Boys**										
GCKRrs1260326	CC	62 (20.8)	25 (14.5)		Reference			Reference		
	CT	162 (54.4)	97 (56.1)		1.49	0.88 to 2.52	0.142	1.4	0.79 to 2.48	0.247
	TT	74 (24.8)	51 (29.5)		1.71	0.95 to 3.07	0.073	1.57	0.82 to 2.98	0.173
				Additive	1.28	0.97 to 1.71	0.085	1.23	0.90 to 1.69	0.192
				Recessive	1.27	0.83 to 1.93	0.271	1.21	0.76 to 1.93	0.428
				Dominant	1.56	0.94 to 2.58	0.088	1.45	0.84 to 2.51	0.186
GCKRrs780094	CC	66 (22.1)	27 (15.6)		Reference			Reference		
	CT	170 (56.9)	99 (57.2)		1.42	0.85 to 2.38	0.176	1.34	0.77 to 2.32	0.305
	TT	63 (21.1)	47 (27.2)		1.82*	1.02 to 3.28	0.044	1.65	0.87 to 3.14	0.126
				Additive	1.34*	1.01 to 1.79	0.045	1.28	0.93 to 1.76	0.128
				Recessive	1.4	0.90 to 2.16	0.132	1.33	0.82 to 2.15	0.253
				Dominant	1.53	0.94 to 2.51	0.09	1.42	0.83 to 2.42	0.2
**Girls**										
GCKRrs1260326	CC	110 (26.8)	16 (19.0)		Reference			Reference		
	CT	198 (48.3)	38 (45.2)		1.32	0.70 to 2.48	0.388	1.47	0.74 to 2.91	0.267
	TT	102 (24.9)	30 (35.7)		2.02*	1.04 to 3.93	0.038	2.29*	1.11 to 4.73	0.026
				Additive	1.44*	1.03 to 2.00	0.032	1.52*	1.06 to 2.17	0.022
				Recessive	1.68*	1.02 to 2.76	0.042	1.75*	1.02 to 3.00	0.041
				Dominant	1.56	0.87 to 2.80	0.138	1.74	0.92 to 3.30	0.091
GCKRrs780094	CC	116 (28.2)	16 (19.0)		Reference			Reference		
	CT	199 (48.4)	41 (48.8)		1.49	0.80 to 2.78	0.206	1.7	0.87 to 3.32	0.124
	TT	96 (23.4)	27 (32.1)		2.04*	1.04 to 4.00	0.039	2.28*	1.09 to 4.75	0.028
				Additive	1.42*	1.02 to 1.98	0.038	1.49*	1.04 to 2.13	0.029
				Recessive	1.55	0.93 to 2.59	0.091	1.58	0.91 to 2.74	0.103
				Dominant	1.67	0.93 to 3.00	0.086	1.88	1.00 to 3.56	0.052

Model 1: unadjusted analysis for possible confounding factors, Model 2: adjusted for age, body mass index, waist circumference, blood pressure, fasting plasma glucose, triglycerides, high density lipoprotein cholesterol, and gender (only in overall group).

OR: odds ratio; CI: confidence interval; NUA: normouricemia; HUA: hyperuricemia.

*Statistically significant difference.

Table 5 presents the results of logistic regression analyses of the metabolic traits concerning the *GCKR* variants of rs1260326 and rs780094. The participants with the TT genotype showed a higher incidence of low HDL-C levels than those with the C allele of rs1260326 after adjusting for confounders, with an OR of 1.56 (95% CI 1.09–2.24, *p* = 0.016). Apart from rs1260326, participants with the TT genotype of rs780094 also had lower HDL-C levels than those with the C allele after adjusting for confounders, with an OR of 1.48 (95% CI 1.02–2.14, *p* = 0.041). However, a significant association of high TG levels was noted only in an additive model (TT vs. CT vs. CC: OR = 1.73, 95% CI 1.03–2.90, *p* = 0.040) in *GCKR* rs1260326.

**Table 5:** Logistic regression analyses of metabolic traits in Taiwanese adolescents with different genotypes of the GCKR gene rs1260326 and rs780094.

GCKRrs1260326		Model 1			Model 2		
		OR	95% CI	p value	OR	95% CI	p value
Met_WC	CT vs. CC	1.00	0.66–1.51	0.998	1.05	0.70–1.59	0.808
	TT vs. CC	1.35	0.86–2.11	0.194	1.39	0.89–2.18	0.153
	Additive model	1.18	0.94–1.48	0.161	1.19	0.95–1.50	0.131
	TT vs (CC + CT)	1.35	0.95–1.90	0.091	1.34	0.95–1.89	0.096
	(CT + TT) vs CC	1.11	0.76–1.64	0.585	1.17	0.79–1.72	0.442
Met_TG	CT vs. CC	1.75	0.58–5.28	0.324	1.64	0.54–4.99	0.381
	TT vs. CC	3.01	0.98–9.29	0.055	2.91	0.94–9.00	0.064
	Additive model	1.73	1.04–2.90*	0.036	1.73	1.03–2.90*	0.040
	TT vs (CC + CT)	1.98	0.99–3.98	0.055	2.00	0.99–4.03	0.052
	(CT + TT) vs CC	2.17	0.76–6.23	0.150	2.07	0.72–5.95	0.179
Met_HDL-C	CT vs. CC	1.49	0.94–2.37	0.093	1.34	0.83–2.15	0.226
	TT vs. CC	2.05	1.25–3.38*	0.005	1.93	1.17–3.21*	0.011
	Additive model	1.42	1.12–1.81*	0.004	1.40	1.09–1.79*	0.008
	TT vs (CC + CT)	1.54	1.08–2.19*	0.017	1.56	1.09–2.24*	0.016
	(CT + TT) vs CC	1.67	1.08–2.60*	0.022	1.53	0.98–2.40	0.064
Met_BP	CT vs. CC	1.43	0.88–2.31	0.147	1.34	0.82–2.18	0.238
	TT vs. CC	1.55	0.92–2.64	0.102	1.47	0.86–2.51	0.158
	Additive model	1.22	0.95–1.58	0.117	1.20	0.92–1.55	0.174
	TT vs (CC + CT)	1.20	0.82–1.76	0.355	1.19	0.80–1.75	0.392
	(CT + TT) vs CC	1.47	0.93–2.33	0.100	1.38	0.87–2.20	0.170
Met_FPG	CT vs. CC	1.04	0.67–1.63	0.857	1.01	0.64–1.58	0.967
	TT vs. CC	0.89	0.53–1.50	0.665	0.87	0.52–1.46	0.600
	Additive model	0.94	0.73–1.21	0.644	0.93	0.72–1.20	0.582
	TT vs (CC + CT)	0.87	0.57–1.31	0.494	0.86	0.57–1.30	0.488
	(CT + TT) vs CC	0.99	0.65–1.52	0.963	0.96	0.63–1.48	0.856

GCKRrs780094		Model 1			Model 2		
		OR	95% CI	p value	OR	95% CI	p value
Met_WC	CT vs. CC	1.03	0.69–1.54	0.887	1.08	0.72–1.63	0.698
	TT vs. CC	1.49	0.95–2.33	0.084	1.53	0.97–2.40	0.068
	Additive model	1.23	0.98–1.55	0.073	1.25	0.99–1.57	0.060
	TT vs (CC + CT)	1.46	1.03–2.07*	0.035	1.44	1.01–2.05*	0.042
	(CT + TT) vs CC	1.17	0.80–1.71	0.432	1.22	0.83–1.79	0.314
Met_TG	CT vs. CC	2.14	0.72–6.37	0.171	2.02	0.68–6.02	0.209
	TT vs. CC	2.74	0.86–8.73	0.089	2.67	0.84–8.53	0.097
	Additive model	1.51	0.93–2.58	0.090	1.55	0.93–2.59	0.096
	TT vs (CC + CT)	1.53	0.74–3.19	0.255	1.56	0.75–3.26	0.236
	(CT + TT) vs CC	2.33	0.81–6.68	0.116	2.22	0.77–6.38	0.140
Met_HDL-C	CT vs. CC	1.53	0.98–2.40	0.063	1.38	0.87–2.18	0.169
	TT vs. CC	1.94	1.18–3.19*	0.009	1.86	1.12–3.09*	0.016
	Additive model	1.38	1.08–1.75*	0.009	1.36	1.06–1.75*	0.015
	TT vs (CC + CT)	1.43	0.99–2.05	0.056	1.48	1.02–2.14*	0.041
	(CT + TT) vs CC	1.66	1.08–2.55*	0.022	1.52	0.98–2.36	0.060
Met_BP	CT vs. CC	1.51	0.95–2.42	0.085	1.43	0.89–2.30	0.145
	TT vs. CC	1.54	0.90–2.63	0.114	1.48	0.86–2.53	0.157
	Additive model	1.22	0.94–1.57	0.128	1.20	0.92–1.55	0.173
	TT vs (CC + CT)	1.14	0.77–1.70	0.519	1.14	0.76–1.71	0.520
	(CT + TT) vs CC	1.52	0.97–2.39	0.069	1.44	0.91–2.28	0.117
Met_FPG	CT vs. CC	0.92	0.60–1.42	0.710	0.89	0.58–1.38	0.607
	TT vs. CC	0.84	0.50–1.40	0.836	0.82	0.49–1.38	0.452
	Additive model	0.91	0.71–1.18	0.496	0.91	0.70–1.17	0.451
	TT vs (CC + CT)	0.88	0.58–1.35	0.569	0.89	0.58–1.36	0.579
	(CT + TT) vs CC	0.89	0.59–1.35	0.594	0.87	0.58–1.31	0.506

Model 1: unadjusted analysis for possible confounding factors; Model 2: adjusted for age, sex.

Met: metabolic trait; WC: waist circumstance; TG: triglyceride; HDL-C: high-density lipoprotein cholesterol; BP: blood pressure; FPG: fasting plasma glucose.

CI, confidence interval; OR, odds ratio.

*Statistically significant differences.

## Discussion

Hyperuricemia is closely associated with several chronic disorders. Although, to the best of our knowledge, no prospective studies have revealed any association between the lower levels of UA and prevention or risk reduction of CVD, patients with hyperuricemia should still be monitored for CVD. Recently, a study involving the Han Chinese population revealed that hyperuricemia is an early-onset metabolic disorder that occurs earlier than the occurrence of other symptoms associated with the risk of CVDs^44^. Moreover, this previous Mendelian randomization (MR) study disclosed the causal role of hyperuricemia in CVD development^44^. Conversely, other MR studies did not support this finding. In a two-sample MR study, the serum UA levels were not significantly associated with the risk of coronary artery disease (CAD) in European patients with diabetes^45^. Another MR study did not support the causal function of the elevated serum UA levels in premature CAD in the Mexican population^46^. This causal relationship remains unclear because of the inconsistent results of the previous studies. Various ethnic population characteristics may be associated with these differences. However, the apparent pleiotropic effect of the *GCKR* variants may influence the CVD risk by affecting the other risk factors for CVD. We found that the participants with specific *GCKR* polymorphisms showed a higher risk for hyperuricemia and other metabolic traits on the basis of gender differences in Taiwanese adolescents. Participants with this association may be prone to CVD development in the future.

Several previous studies explored the role of the *GCKR* variants in regulating the serum UA levels and/or gout. Most of these studies identified a strong correlation between the genetic variants of the *GCKR* polymorphisms, including rs1260326^10,27–29,42,43^ and rs780094^10,11,26,27,30,38–42^, and hyperuricemia and/or gout development. To the best of our knowledge, our study first demonstrates this association in Taiwanese adolescents. Our findings were similar to those of previous studies conducted in adult populations. Hyperuricemia is also related to insulin resistance^47^ and is considered one of the etiologies of metabolic syndrome^48^, indicating that both diseases share a common genetic background. Because GCK phosphorylates glucose to form glucose 6-phosphate and thereby modulates hepatic glucose disposal and activates hepatic lipogenesis^21,22,49^, the close relationship between the *GCKR* variants and insulin resistance and/or glucose intolerance was explored in a previous study^50^. We analyzed our data to explore the association between the *GCKR* variants and metabolic syndrome components. Low serum HDL-C levels were more prevalent in participants with the T allele than in those with the C allele of rs780094 in Taiwanese adolescents. Apart from rs780094, the low serum HDL-C levels were more prevalent in participants with the TT genotype of rs1260326 than in those with the C allele. These results are similar to those of a recent retrospective cohort study^51^ but different from the findings of another study^52^ reporting that the T allele of rs780094 in white participant is associated with higher HDL-C levels. However, the difference was not statistically significant in African Americans. Most previous studies have confirmed the association between the T allele or T carrier genotypes of the *GCKR* variants and metabolic traits, particularly higher TG and fasting plasma glucose levels^25,53^. However, the exact mechanism underlying the effect of *GCKR* on the serum UA levels remains unknown. Several possible mechanisms have been proposed in the literature. Glucose metabolic abnormality related to *GCK/GCKR* expression leads to obesity, an important contributor of hyperuricemia development^54^. A previous study presented a hypothesis that *GCKR* modulates hepatic inorganic phosphate homeostasis and induces the subsequent elevation of the serum UA levels^55^. Hyperinsulinemia might cause a significant decrease in urinary UA excretion by increasing UA reabsorption in the kidney, thereby inducing further hyperuricemia^56^. Another study reported that a *GCKR* variant was associated with lower fractional excretion of UA through the increase of UA reabsorption in the proximal renal tubules^28^. Other studies suggested that the *GCKR* variants influenced metabolite levels in the glycolytic pathway, thereby altering renal UA excretion^57^.

Not all studies, however, reported that the *GCKR* variants influence the serum UA levels^32,43^. The discrepancy in results may be attributed to several factors. First, the study population and differences in the minor allele frequency between ethnic populations may play a role. Second, limited sample sizes with limited power and inadequate effect sizes of the risk variants may also influence study results. Furthermore, the possible interaction between *GCKR* polymorphism and lifestyle habits, such as drinking or eating habits, may also play a role. An European study showed that alcohol drinking by individuals with *GCKR* rs780094 strongly influenced the risk of hyperuricemia compared with that noted in the case of no alcohol consumption^39^. This is consistent with the hypothesis that *GCKR* controls gout risk through its physiological role in glycolysis, presumably resulting in increased endogenous UA production. However, this gene-alcohol interaction was not observed in another Japanese study that focused on the same polymorphism^40^. Genetic differences between distinct ethnic populations may also attribute to this discrepancy.

Interestingly, our study showed a gender-based difference in the *GCKR* variants concerning the influence on the serum UA levels (T allele). Only girls with the TT genotypes of the two SNPs (rs780094 and rs1260326) had a higher risk for hyperuricemia than those with the CC genotypes after adjusting for confounders. The data are limited regarding gender specification. No sexual dimorphism was observed in the effect of *GCKR* on the serum UA levels in a Japanese study^40^ or a large-scale meta-analysis^10^. Recently, a study demonstrated significant gender-specific differences in the effect of the *GCKR* variant rs1260326; however, the gender-specific effect was not observed in the more stringent genome-wide study investigating the effects of SNP on the UA levels^58^. The differences between the results remain unclear; however, the study population, ethnic factor, and sex hormone may contribute to these differences. The prevalence of hyperuricemia or gout differed between the genders, which might be attributed to the uricosuric effect of the female sex hormone on the serum UA level regulation in animals and humans^59–61^. *SLC2A9* was also recognized as an apical UA transporter in the renal tubules^62^, which might increase the fractional excretion of UA and decrease the serum UA levels^63^. Significant sexual dimorphism was also observed in the genetic variants of *SLC2A9* in the serum UA level regulation, implying a gender effect^10,40^. However, the exact mechanisms underlying the effect of gender differences on the serum UA levels remain unclear^64^.

The major strength of the present study is that we explored the association between the *GCKR* variants and serum UA levels in Taiwanese adolescents on the basis of gender differences. However, there are some limitations of our study that need to be addressed. First, the definition of hyperuricemia varies between adults and adolescents. Therefore, our findings may not be applicable to adult populations. Second, several factors affect the serum UA levels, such as lifestyle patterns. Although the adolescents are assumed to have similar lifestyles, we did adjust for the factor related to alcohol consumption. Third, the relatively small numbers of our study participants should be considered. Fourth, hormone profiles also influence the serum UA levels; we did not obtain hormone profiles in our analysis. Furthermore, complex diseases may be induced by multiple genetic interactions. Finally, the pleiotropy of the genetic functions may explain the failure to replicate previous reports.

## Conclusions

This study showed that *GCKR* polymorphisms may regulate the serum UA levels on the basis of gender differences in Taiwanese adolescents. However, larger studies are warranted in the future to confirm this association.

## Supplementary Information

Supplementary Table 1.

Supplementary Table 2.
