# Supplementary material for: Association between glucokinase regulator gene polymorphisms and serum uric acid levels in Taiwanese adolescents
Source: Sci Rep. 2022 Apr 1;12:5519. doi: 10.1038/s41598-022-09393-5 (PMC8975867; doi:10.1038/s41598-022-09393-5)
Supplement: Supplementary file 1 — Supplementary Table 1. [file 41598_2022_9393_MOESM1_ESM.docx]

Supplementary Table 1. GCKR Alleles and hyperuricemia frequencies in Taiwanese adolescents

|  | NUA n (%) | HUA n (%) | p value |
| --- | --- | --- | --- |
| All |  |  |  |
| GCKRrs1260326 |  |  | 0.004* |
| C | 704(49.7%) | 217(42.2%) |  |
| T | 712(50.3%) | 297(57.8%) |  |
| GCKRrs780094 |  |  | 0.003* |
| C | 733(51.6%) | 226(44.0%) |  |
| T | 687(48.4%) | 288(56.0%) |  |
|  |  |  |  |
| Boys |  |  |  |
| GCKRrs1260326 |  |  | 0.102 |
| C | 286(48.0%) | 147(42.5%) |  |
| T | 310(52.0%) | 199(57.5%) |  |
| GCKRrs780094 |  |  | 0.063 |
| C | 302(50.5%) | 153(44.2%) |  |
| T | 296(49.5%) | 193(55.8%) |  |
|  |  |  |  |
| Girls |  |  |  |
| GCKRrs1260326 |  |  | 0.028* |
| C | 418(51.0%) | 70(41.7%) |  |
| T | 402(49.0%) | 98(58.3%) |  |
| GCKRrs780094 |  |  | 0.034* |
| C | 431(52.4%) | 73(43.5%) |  |
| T | 391(47.6%) | 95(56.5%) |  |

NUA: normal UA; HUA: hyperuricemia

*Statistically significant differences, p < 0.05
