# Supplementary material for: Association between glucokinase regulator gene polymorphisms and serum uric acid levels in Taiwanese adolescents
Source: Sci Rep. 2022 Apr 1;12:5519. doi: 10.1038/s41598-022-09393-5 (PMC8975867; doi:10.1038/s41598-022-09393-5)
Supplement: Supplementary file 2 — Supplementary Table 2. [file 41598_2022_9393_MOESM2_ESM.docx]

Supplementary Table 2. GCKR genotypes and hyperuricemia frequencies in Taiwanese children

|  | NUA n (%) | HUA n (%) | p value |
| --- | --- | --- | --- |
| All |  |  |  |
| GCKRrs1260326 |  |  | 0.010* |
| CC | 172 (24.3) | 41 (16.0) |  |
| CT | 360 (50.8) | 135 (25.5) |  |
| TT | 176 (24.9) | 81 (31.5) |  |
| GCKRrs780094 |  |  | 0.007* |
| CC | 182 (25.6) | 43 (16.7) |  |
| CT | 369 (52.0) | 140 (54.5) |  |
| TT | 159 (22.4) | 74 (28.8) |  |
|  |  |  |  |
| Boys |  |  |  |
| GCKRrs1260326 |  |  | 0.188 |
| CC | 62(20.8%) | 25(14.5%) |  |
| CT | 162(54.4%) | 97(56.1%) |  |
| TT | 74(24.8%) | 51(29.5%) |  |
| GCKRrs780094 |  |  | 0.13 |
| CC | 66(22.1%) | 27(15.6%) |  |
| CT | 170(56.9%) | 99(57.2%) |  |
| TT | 63(21.1%) | 47(27.2%) |  |
|  |  |  |  |
| Girls |  |  |  |
| GCKRrs1260326 |  |  | 0.088 |
| CC | 110(26.8%) | 16(19.0%) |  |
| CT | 198(48.3%) | 38(45.2%) |  |
| TT | 102(24.9%) | 30(35.7%) |  |
| GCKRrs780094 |  |  | 0.112 |
| CC | 116(28.2%) | 16(19.0%) |  |
| CT | 199(48.4%) | 41(48.8%) |  |
| TT | 96(23.4%) | 27(32.1%) |  |

NUA: normal UA; HUA: hyperuricemia

*Statistically significant differences, p < 0.05
